# Elevated Serum PCT in Septic Shock With Endotoxemia Is Associated With a Higher Mortality Rate

**DOI:** 10.1097/MD.0000000000001085

**Published:** 2015-07-13

**Authors:** Barbara Adamik, Jakub Smiechowicz, Dominika Jakubczyk, Andrzej Kübler

**Affiliations:** From the Department of Anaesthesiology and Intensive Therapy, Wroclaw Medical University, Wroclaw, Poland (BA, DJ, AK) and Department of Anaesthesiology and Intensive Therapy, Wroclaw University Hospital, Wroclaw, Poland (JS).

## Abstract

To examine the effect of endotoxemia on the procalcitonin (PCT) serum levels and mortality rates of adult patients with septic shock diagnosed on the day of admission to the intensive care unit (ICU).

A retrospective observational study was performed over a 2-year period. Levels of PCT were compared for septic shock patients with and without endotoxemia on admission to the ICU. Endotoxemia was identified with an Endotoxin Activity Assay.

One hundred fifty-seven patients with septic shock were enrolled into the study. Group 1 consisted of patients with elevated endotoxin activity (EA) (n = 95, EA = 0.57 endotoxin activity unit [EAU] [0.46–0.67]) and Group 2 consisted of patients with low EA (n = 62, EA = 0.27 EAU [0.17–0.36]). Acute Physiology And Chronic Health Evaluation II (APACHE II) score and SOFA score were similar in both groups (APACHE II = 23 [16–29] and 19 [16–25]; Sequential Organ Failure Assessment [SOFA] = 10 [7–13] and 11 [8–12] in Groups 1 and 2, respectively) (nonsignificant). The PCT level was 6 times higher in Group 1 than in Group 2 (19.6 ng/mL vs. 3.1 ng/mL, *P* < 0.001). There was a strong correlation between EA and serum PCT (*P* < 0.001, R = 0.5). The presence of endotoxemia on admission to the ICU was associated with an increased mortality rate: 52% in the group of patients with endotoxemia and 25% in the group without endotoxemia. EA in survivors was 0.39 EAU (0.26–0.57) and 0.53 EAU (0.4–0.61) in nonsurvivors (*P* = 0.004). The median PCT level in survivors was 6.7 ng/mL (2.3–28.0), compared with 16.7 ng/mL (5.3–31.0) in nonsurvivors (*P* = 0.04).

This observational study revealed that endotoxemia in patients with septic shock on admission to the ICU was frequently found and was associated with an elevated PCT level and a high mortality rate. Endotoxemia was a common occurrence in patients with septic shock, regardless of the infecting microorganism.

## INTRODUCTION

Procalcitonin (PCT), a precursor of the hormone calcitonin, is synthesized physiologically by thyroid C cells. In normal human serum, the PCT level is low.^1^ Increased PCT levels were first reported by Assicot et al^2^ to be associated with various bacterial infections. In an animal model of sepsis, in which bacterial infection was induced by implanting *Escherichia coli* pellets, increased calcitonin mRNA expression as well as elevated PCT plasma level was observed in various tissues that are not usually viewed as being endocrine.^3^ Systemic PCT secretion is a component of the inflammatory response, and it is particularly evident in bacterial infections.^4,5^ An elevated serum PCT level was reported in the course of a severe bacterial infection in patients who had previously undergone a total thyroidectomy.^2^ Systemic changes are triggered by bacterial components, such as endotoxin, peptidoglycan, lipoteichoic acid, and lipoprotein; these components stimulate the production of many mediators of inflammation. There is evidence that gram-negative bacteremias cause higher rises in PCT than gram-positive bacteremias.^6^ The predictive value of PCT for clinical outcomes has been extensively studied in groups of critically ill patients and has yielded variable results. The damaging effect of PCT in the pathogenesis of sepsis has been reported based on animal studies.^7,8^ Administering PCT to septic animals had several toxic effects and greatly increased mortality. In contrast, an anti-inflammatory effect of PCT was reported in several studies.^9–11^ The capability of PCT to neutralize bacterial endotoxin and to reduce the tumor necrosis factor (TNF)-alpha level was demonstrated in a model of human peripheral blood mononuclear cells.^9^

In this study, the effect of endotoxemia was evaluated on the PCT serum level and the mortality rate of patients with septic shock on the day of admission to the intensive care unit (ICU). An unresolved question is whether PCT is a harmful biomarker or a mediator that directly neutralizes bacterial lipopolysaccharide (LPS). Understanding the role of PCT in bacterial infection is crucial, because of the significant impact of endotoxemia on the PCT level and mortality in patients with septic shock, as was observed in this study.

## SUBJECTS AND METHODS

### Study Group

The study design was a single-center retrospective observational analysis of all adult patients with a diagnosis of septic shock admitted to the 25-bed mixed ICU in a 996-bed tertiary-care University Hospital from January 2012 to December 2013. The Ethics Committee of the Wroclaw Medical University approved the study protocol (KB-692/2014). The need for informed consent was waived due to the retrospective, observational nature of the study. Included in the study were adult patients with septic shock, according to the consensus definition of the American College of Chest Physicians/Society of Critical Care Medicine Consensus Conference (ACCP/SCCM),^12^ where the PCT level and endotoxin activity (EA) were measured within the first 24 h after admission to the ICU. All patients in the study received standard treatment for septic shock according to the Surviving Sepsis Campaign guidelines.^13^ The baseline severity of the illness was quantified with the Acute Physiology and Chronic Health Evaluation II (APACHE II) score and the degree of organ dysfunction was assessed with a Sequential Organ Failure Assessment (SOFA) score on admission to the ICU. Demographic data, microbiology results, and routine parameters, such as white blood cell (WBC) count, C-reactive protein (CRP) level, creatinin and bilirubin level, and coagulation parameters (APTT, activated partial thromboplastin time; PT, prothrombin time) were also recorded.

### PCT and Endotoxin Measurement

Serum concentrations of PCT were determined by an immunoluminometric assay (LUMI test PCT) (BRAHMS Diagnostica, Berlin, Germany) and the detection limit was 0.05 ng/mL. EA was measured in a whole blood sample drawn from an intravenous catheter to a tube with ethylenediaminetetraacetic acid (EDTA). Endotoxemia was identified with a Endotoxin Activity Assay (EAA, Spectral Diagnostics, Inc., Toronto, Canada), commercially available, CE, in vitro diagnostic (IVD) marked diagnostic test intended for measuring EA in clinical samples. This method is based on the detection of the respiratory burst activity generated in neutrophils. Oxygen radicals generated by primed neutrophils produce luminal chemiluminescence, and the signal is recorded with a luminometer (single tube luminometer Smart Line TL, Berthold Detection Systems GmbH, Pforzheim, Germany). The results are quantitative, expressed in endotoxin activity units (EAU) on a scale from 0 to 1, and they represent the mean value of duplicate analysis from each blood sample.

### Outcomes and Statistical Analysis

Whole blood EA and the PCT serum level were immediately measured in patients with septic shock on admission to the ICU. In accordance with the manufacturer's information, endotoxemia was defined as EA ≥ 0.4 EAU. Patients with septic shock on admission to the ICU were divided into 1 of 2 groups according to the EA results: Group 1 with elevated EA (EA ≥ 0.4 EAU) and Group 2 with low EA (EA < 0.4 EAU). The ICU mortality rate was recorded and the ICU length of stay was counted until discharge from the ICU or death. Only patients with septic shock diagnosed on the admission to the ICU were included. This was done in order to obtain the most homogenous group of ICU patients.

These data were analyzed with Statistica 10 (StatSoft, Inc., Tulsa, OK). Patients with missing data were excluded from the analysis. The distribution of the variables was not normal, based on a Shapiro–Wilk test. Therefore, statistical analysis of the data was performed using nonparametric techniques. Continuous variables are presented as medians with the 25th and 75th percentiles. The Mann–Whitney *U* test was used to compare differences between 2 independent groups. The relationship between the EA and other parameters was assessed with a Spearman rank correlation test. Categorical variables were analyzed using a Pearson χ^2^ test. Survival analysis of time to death was performed using the Kaplan–Meier curve and log-rank test. Statistical significance was determined as *P* < 0.05.

## RESULTS

One hundred fifty-seven patients diagnosed with septic shock were enrolled into the study. All patients had their EA level measured on admission to the ICU and based on the value of the EA, patients were placed into Group 1 (N = 95; EA = 0.57 EAU [0.46–0.67]) or Group 2 (N = 62; EA = 0.27 EAU [0.17–0.36]). The severity of the clinical status as estimated by the APACHE II and SOFA scores was similar for both groups (APACHE II = 23 [16–29] and 19 [16–25]; SOFA = 10 [7–13] and 11 [8–12] in Groups 1 and 2, respectively) and no statistically significant difference was observed. The PCT level was 6 times higher in Group 1 than in Group 2 (19.6 ng/mL vs. 3.1 ng/mL, *P* < 0.001). The median serum creatinine value in patients with endotoxemia was statistically greater (*P* = 0.03) than in patients without endotoxemia and renal replacement therapy was instituted in 64% of patients in Group 1 and in 45% of patients in Group 2 (*P* = 0.03). There were no differences in the CRP, bilirubin, PLT count, WBC, and coagulation parameters between the study groups. The baseline characteristics of the study group are presented in Table 1.

**TABLE 1:**
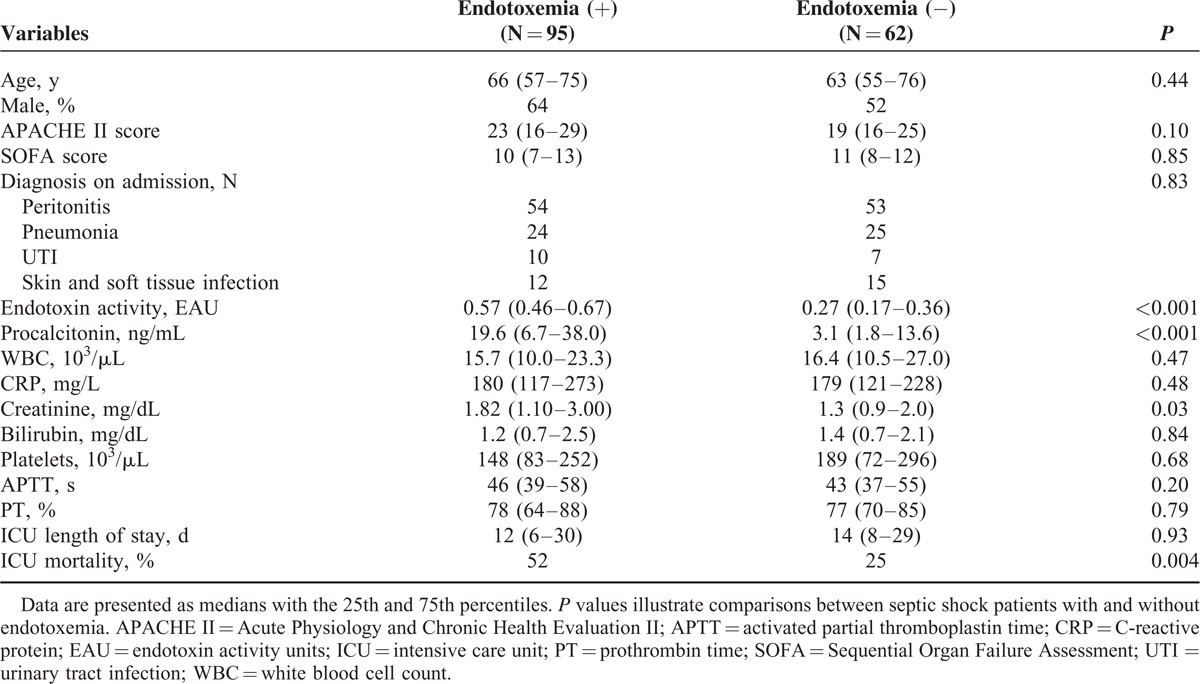
Demographic and Baseline Characteristics of Patients With Septic Shock

### PCT, Endotoxemia, and Pathogen Detection

On admission to the ICU, serum PCT levels were higher than 0.5 ng/L in 95% of patients with septic shock; in 18% of patients <2 ng/mL, in 33% ≥2 to <10 ng/mL, and in 49% PCT ≥10. A higher level of serum PCT was associated with a significant decrease in the survival rate (*P* = 0.05) (Table 2). Endotoxemia, defined as EA ≥ 0.4 EAU, was present in the majority of patients (60%). There was a strong correlation between EA and serum PCT (*P* < 0.001, R = 0.5).

**TABLE 2:**
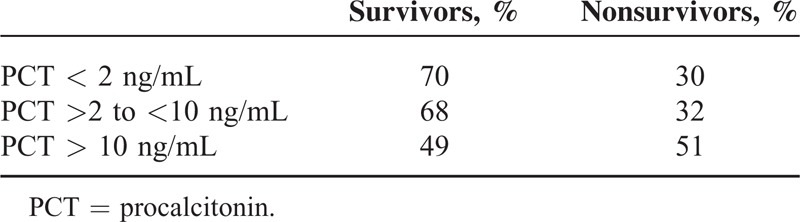
Distribution of Serum Procalcitonin Levels (PCT) in 3 Reference Ranges in Survivors (N = 64) and Nonsurvivors (N = 93) (*P* = 0.05; Pearson χ^2^)

### Pathogen Detection

**FIGURE 1:**
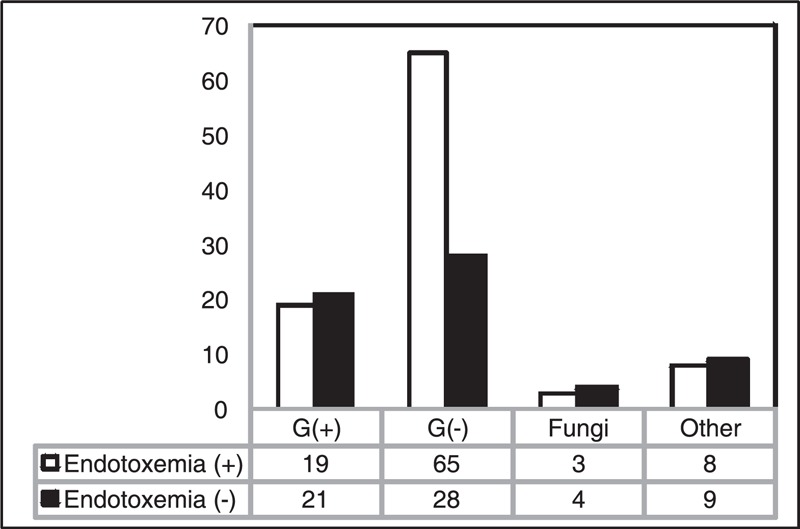
The distribution of pathogens identified in patients with septic shock and endotoxemia (N = 95) and without endotoxemia (N = 62) (*P* = 0.002).

In patients with endotoxemia, a gram-negative pathogen was identified as the primary source of infection leading to septic shock in 68% of patients, a gram-positive in 20%, fungi in 3%, viral in 4%, and unknown in 5%. In patients without endotoxemia, the distribution of pathogens was significantly different (*P* = 0.002): in 45% of patients a gram-negative pathogen was identified, a gram-positive in 34%, fungi in 6%, *Mycoplasma pneumonia* in 3%, viral in 2%, and unknown in 9%. The distribution of identified pathogens in both groups is shown in Figure 1. Bacteriemia was detected more often in the group with endotoxemia, compared to the group without endotoxemia (26 [27%] vs. 8 [13%]; *P* = 0.01).

### Survival Analysis

**FIGURE 2:**
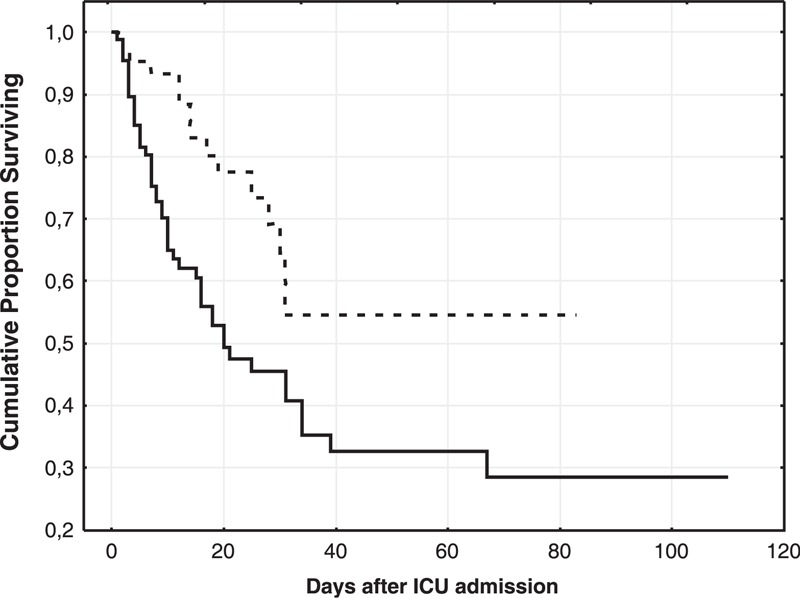
The Kaplan–Meier survival analysis. The solid line represents the group with endotoxemia and the dotted line represents the group without endotoxemia. Log-rank test *P* = 0.001.

The ICU mortality of the whole study group of patients with septic shock was 41%. The Kaplan–Meier survival analysis of time to death showed that there was statistical significance between groups with and without endotoxemia (*P* = 0.001, log-rank test) (Figure 2). The presence of endotoxemia on admission to the ICU was associated with an increased mortality rate: 52% in the group with endotoxemia and 25% in the group without endotoxemia. Median EA in survivors was 0.39 EAU (0.26–0.57) and in nonsurvivors was 0.53 EAU (0.4–0.61) (*P* = 0.004). Median PCT level in survivors was 6.7 ng/mL (2.3–28.0), compared to 16.7 ng/mL (5.3–31.0) in nonsurvivors (*P* = 0.04).

## DISCUSSION

The study revealed that endotoxemia in patients with septic shock on admission to the ICU was frequently found and was associated with an elevated PCT level. The mortality rate was 2 times higher in patients with increased EA.

Endotoxin, an LPS present in the outer cell membrane of gram-negative bacteria, is considered to be the principal mediator involved in the development of septic shock. LPS can be recognized by the toll-like receptor 4 (TLR4), a transmembrane receptor expressed in different immune cells, such as neutrophils, monocytes, macrophages, and dendritic cells.^14^ On activation, these cells produce inflammatory mediators, eg, the TNF and interleukins (IL-1beta, IL-6, IL-8, IL-10), which leads to the induction of a variety of inflammatory processes and may contribute to the development of major organ failure in the course of sepsis.^15,16^ Previous research indicates that endotoxemia is common in populations of ICU patients on the day of admission. In an MEDIC study, in a heterogeneous population of ICU patients, 57% had elevated EA.^17^ In another study, in which EA in a population of ICU surgical patients was analyzed, the baseline EA level was elevated in 34% of patients.^18^ Our data add to the existing research on the prevalence of endotoxemia in patients diagnosed with septic shock; the majority of studied patients had an elevated EA level on admission to the ICU. Mortality in the group with septic shock and endotoxemia was 2 times higher than in patients without endotoxemia. Endotoxin is a powerful inducer of proinflammatory cytokine production, an activator of inducible nitric oxide synthase, the complement system, and coagulation processes. All of these can contribute to the development of a systemic inflammatory reaction and could account for the observed higher mortality rate in the studied population. The effect on mortality rates of endotoxemia detected in different study groups on admission to the ICU has been described before.^19,20^ In a Yaguchi study performed on a heterogeneous population of ICU patients, the highest mortality rate was reported in patients with endotoxemia, with EA > 0.6 EAU.^20^ In our study, the Kaplan–Meier survival analysis revealed a significantly higher mortality rate in patients with endotoxemia, when EA ≥ 0.4 EAU.

Endotoxemia in septic shock could be a manifestation of an exogenous infection or a result of LPS translocation. In this study in patients with endotoxemia, a gram-negative pathogen as the primary source of infection in septic shock was detected in the majority of patients; however, some patients with an infection other than gram-negative also had elevated EA. Endotoxemia in these patients could be the result of the translocation of gram-negative bacteria or LPS alone through the damaged intestinal wall to normally sterile tissues. Regional hypoperfusion and ischemia of the intestinal mucosa are factors that can promote translocation in septic shock. These data are consistent with that of previously reported studies.^20,21^ Yaguchi et al observed a low rate of gram-negative organisms in patients with sepsis and a low level of EA; this rate increased significantly to 70% in patients with elevated EA.^20^

The serum elevation of PCT recorded in the majority of patients with septic shock in our study might suggest that there had been previous stimulation of the inflammatory cytokine cascade and indicate a dependency of the PCT level on the release of the inflammatory mediators. It has been previously demonstrated that the administration of PCT alone does not initiate inflammatory cytokine release, either in healthy or septic animals, which suggests that PCT is a secondary mediator that requires other mediators to initiate production.^22–24^ In fact, it was observed that an injection of a pro-inflammatory cytokine—TNF-alpha—was associated with as high as a 25-fold increase in the PCT level 12 h after TNF-alpha stimulation.^24^ Structural studies revealed that there are 2 LPS binding sequences present in the PCT molecule and PCT might directly bind to a lipid A in the LPS molecule and participate in endotoxin clearance.^25,26^ Matera et al^9^ examined the effect of PCT on the release of cytokines by human peripheral blood mononuclear cells and demonstrated that PCT neutralized LPS molecules and significantly decreased LPS reactivity in vitro. The decrease in the LPS level had an effect on cytokine production. A subsequent reduction in TNF-alpha, IL-10, and the mononuclear cell targeting chemokine (MCP-1) in LPS-stimulated cells was observed after incubation with PCT. In our study, the PCT level was high in patients with an elevated EA at the onset of septic shock. Considering the results of in vitro studies, it is possible that, at least in part, PCT in patients with septic shock and endotoxemia was produced and released to bind and to neutralize endotoxins circulating in the blood. It should be also noted that serum creatinine was significantly higher in the group with endotoxemia. Endotoxin induces cellular damage and is considered to be one of the factors behind the development of acute renal failure during sepsis.^27^ An induction of significant pro-apoptotic mechanisms by LPS in various renal cell types, including tubule cells and glomerular endothelial cells, has been observed and can effect renal function by decreasing the number of cells in the organ or inhibiting communication between cells.^28,29^ This potential pro-apoptotic mechanism leading to renal injury in sepsis can explain the finding in our study that the requirement for renal replacement therapy was higher in the group with endotoxemia. Admittedly, the observational and retrospective nature of this study was a limitation, and only patients with septic shock diagnosed on the admission to the ICU were included. This was done in order to obtain the most homogenous group of ICU patients.

## CONCLUSIONS

The following are the conclusions of this study.

1. This study shows that endotoxemia is common in patients with septic shock.
2. Elevated EA can be detected in critically ill patients regardless of the infecting microorganism.
3. Endotoxemia is accompanied by a high PCT level in septic shock. A strong correlation was observed between EA and serum PCT (*P* < 0.001, R = 0.5).
4. The elevation of PCT level is associated with an increased mortality of patients with septic shock.
5. Endotoxemia is associated with an increased ICU mortality in patients with septic shock.
